# Myosin Light Chain 9/12 Regulates the Pathogenesis of Inflammatory Bowel Disease

**DOI:** 10.3389/fimmu.2020.594297

**Published:** 2021-01-29

**Authors:** Masaya Yokoyama, Motoko Y. Kimura, Toshihiro Ito, Koji Hayashizaki, Yukihiro Endo, Yangsong Wang, Ryoji Yagi, Tomoo Nakagawa, Naoya Kato, Hisahiro Matsubara, Toshinori Nakayama

**Affiliations:** ^1^Department of Immunology, Graduate School of Medicine, Chiba University, Chiba, Japan; ^2^Department of Frontier Surgery, Graduate School of Medicine, Chiba University, Chiba, Japan; ^3^Department of Gastroenterology, Graduate School of Medicine, Chiba University, Chiba, Japan; ^4^Advanced Research and Development Programs for Medical Innovation (AMED-CREST), Japan Agency for Medical Research and Development (AMED), Chiba, Japan

**Keywords:** CD69, Myl9, plasma biomarker, ulcerative colitis, Crohn’s disease

## Abstract

The numbers of patients with inflammatory bowel disease (IBD), such as ulcerative colitis (UC) and Crohn’s disease (CD), have been increasing over time, worldwide; however, the pathogenesis of IBD is multifactorial and has not been fully understood. Myosin light chain 9 and 12a and 12b (Myl9/12) are known as ligands of the CD69 molecule. They create “Myl9 nets” that are often detected in inflamed site, which play a crucial role in regulating the recruitment and retention of CD69-expressing effector cells in inflamed tissues. We demonstrated the strong expression of Myl9/12 in the inflamed gut of IBD patients and mice with DSS-induced colitis. The administration of anti-Myl9/12 Ab to mice with DSS-induced colitis ameliorated the inflammation and prolonged their survival. The plasma Myl9 levels in the patients with active UC and CD were significantly higher than those in patients with disease remission, and may depict the disease severity of IBD patients, especially those with UC. Thus, our results indicate that Myl9/12 are involved in the pathogenesis of IBD, and are likely to be a new therapeutic target for patients suffering from IBD.

## Introduction

Inflammatory bowel disease (IBD), including ulcerative colitis (UC) and Crohn’s disease (CD), is a condition characterized by chronic inflammation of the gastrointestinal tract that causes bloody diarrhea, abdominal pain, and weight loss (1). Over time, the incidence and prevalence of UC and CD have increased worldwide (2, 3). The pathogenesis of IBDs is suggested to be multifactorial, including genetic factors, environmental factors, and immune disorders causing defects in epithelial barriers, thereby resulting in intestinal inflammation (4–7). Immune disorders are known to be deeply involved in the pathogenesis of IBD. CD4 T cells seem to play particularly important roles in the pathogenesis of IBD (8). Indeed, CD4 T cells have been found to be enriched in lesional tissues from IBD patients, and the depletion of CD4 T cells using CD4^+^ cell-depleting antibody (Ab) significantly ameliorated IBD symptoms (9, 10). However, the etiology of these diseases is still largely unknown, and effective treatments are limited.

CD69 is a type-II transmembrane protein that is recognized as a marker of lymphocyte activation. Our series of studies has demonstrated that CD69 is not only a marker of activation but is involved in the pathogenesis of inflammatory diseases, such as airway inflammation (11, 12). In addition, CD69 is reported to be upregulated in the inflamed colon mucosal tissues of IBD patients, and its expression is suppressed upon treatment (13), suggesting that CD69 plays some role in inflammation of the colon. Indeed, we and others have reported that the CD69 expression on lymphocytes regulates inflammation of the colon in various mouse models (14–16). Notably, our previous study shows that CD69 knockout (KO) mice only develop slight dextran sodium sulphate (DSS)-induced colitis, and that the CD69 expression on CD4 T cells plays important roles in the pathogenesis of DSS-induced colitis (14). However, the detailed mechanism underlying the involvement of the CD69 expression is the pathogenesis of IBD is still largely unknown.

We recently found that myosin light chain (Myl)9, Myl12a, and Myl12b (Myl9/12) are functional ligands for CD69 (17). Myl9 appears to be produced by activated platelets and forms net-like structures named “Myl9 nets” inside blood vessels at inflammatory sites. These Myl9 nets are likely to be used as a platform for the recruitment and maintenance of CD69-expressing inflammatory cells in inflamed tissues, thereby exacerbating inflammation. We have dubbed this system the “CD69-Myl9 system” (12, 18). Importantly, the blockade of the CD69-Myl9 system by treatment with either anti-CD69 or anti-Myl9/12 Ab ameliorates inflammation, such as airway inflammation (17). Thus, both CD69 and Myl9/12 are involved in the pathogenesis of various inflammatory disorders; however, whether Myl9/12 molecules are involved in the pathogenesis of IBD has been unclear.

We herein report that the expression of Myl9/12 was highly detected in the inflamed guts of mice with DSS-induced colitis as well as in IBD patients. CD69-expressing cells were found to be attached to the Myl9 nets. Furthermore, anti-Myl9/12 Ab treatment ameliorated DSS-induced colitis, suggesting that Myl9/12 Ab may have therapeutic potential in IBD. In addition, we found that the Myl9 levels in the plasma of IBD patients, especially UC patients, were strongly correlated with the disease severity, suggesting that plasma Myl9 levels are a useful new biomarker of the disease activity of IBD.

## Materials and Methods

### Patients and Samples

All IBD patients and healthy volunteers included in this study were recruited by Chiba University Hospital (Chiba, Japan), from October 2017 to March 2020. Patient information was extracted from the patients’ medical records, including the disease characteristics, extent of the disease, endoscopic features, clinical and endoscopic assessments of the disease, and medications (**Table 1**). Surgical samples of the colon were collected from 10 UC patients and blood samples were collected from 47 UC patients, and 11 healthy donors (20 patients with active disease and 27 patients in remission). The status of the UC patients was evaluated using the Mayo score (19, 20). Patients were considered to have active colitis when their Mayo score was ≥3, and to be in remission when their Mayo score was <2 (and each subscore was <2). Regarding CD patients, the tissue samples were surgically collected from 4 CD patients and blood samples were collected from 34 CD patients (9 patients with active disease and 25 patients in remission). Patients with Crohn’s disease were evaluated with the Harvey-Bradshaw index (HBI) (21, 22). Patients were considered to have active Crohn’s disease when their HBI was ≥5, and to be in remission when it was <5. The diagnosis for each patient was confirmed based on clinical characteristics, an endoscopic examination and histological features after the exclusion of infectious diseases, other autoimmune diseases and tumors. The study was approved by the Ethics Committee of the Chiba University Graduate School of Medicine (No. 898). Written informed consent was provided by each participant before the study.

**Table 1:** Demographic data and the clinical features of the enrolled patients.

	Ulcerative colitis (n=47)	Chron’s disease (n=34)
Male n (%)	27(57.4)	26(76.5)
Female n (%)	20(42.6)	8(23.5)
Age, years (mean + SD)	42.47 ± 16.22	40.5 ± 13.67
Disease type, n (%)		
UC proctitis	4(8.5)	
Left-sided colitis	10(21.3)	
Pancolitis	33(70.2)	
CD colitis		3(8.8)
Ileocolitis		19(55.9)
jejunoileitis		11(32.4)
other type		1(2.9)
Disease activity, n (%)		
UC remission	27(57.4)	
Active	20(42.6)	
CD remission		25(73.5)
Active		9(26.5)
Medication, n (%)		
5-ASA	41(87.2)	22(64.7)
Corticosteroid	16(34.0)	11(32.4)
Immunomodulator	17(36.2)	15(44.1)
Calcineurin inhibitor	10(21.3)	
Anti-TNFα	14(29.8	25(73.5)
UST		3(8.8)
VED	1(2.1)	
TOF	4(8.5)	
5-ASA enema	6(12.8)	
Corticosteroid enema	10(21.3)	
Surgery, n (%)	10(21.3)	4(11.8)

SD, standard deviation; 5-ASA, 5 amino-salicylate; UST, Ustekinumab; VED, Vedolizumab; TOF, Tofacitinib.

### Mice

C57BL/6 female mice were purchased from CLEA, Co. (Tokyo, Japan). All mice (6–8 weeks old) were housed under specific-pathogen-free conditions, and all experiments were approved by the Chiba University Review Board for Animal Care.

### DSS-Induced Colitis Model

Colitis was induced in female mice (age, 7 to 8 weeks) by adding 2% DSS (MW 36,000–50,000; MP Biomedicals, Solon, OH, USA) to drinking water for up to 12 days. The body weight, stool consistency, and fecal blood loss were recorded daily. The disease activity index (DAI) was calculated as described in **Supplementary Table 1**. As indicated, we intraperitonially injected anti-Myl9/12 mAb (114-2G9; KAN Research Institute Inc.) or Control isotype Ab (104-6G4; KAN Research Institute Inc.) into mice with DSS-induced colitis every 2 days from day 0 to day 12 (day 0: 200 µg, day 2–12: 100 µg). The colon was fixed in 10% buffered formalin for the histological analysis. Slides of the distal colon were subjected to hematoxylin and eosin (H&E) staining (H&E; Biopathology Institute Co., Ltd.), and then scored in a blinded fashion using the previously published grading system described in **Supplementary Table 2** (23).

### Immunoblotting

For immunoblotting, colon tissue lysates were prepared using RIPA buffer and then subjected to immunoblotting. The antibodies used for the immunoblotting were rabbit anti-Myl9/12 (F6; Abwiz Bio), mouse anti-Tublin-α (NeoMarkers), and subsequent anti-rabbit IgG-HRP and anti-mouse IgG-HRP (GE Healthcare), respectively.

### Immunohistochemistry

For immunohistochemistry (IHC), cryostat colon sections were fixed in 4% paraformaldehyde and, then stained and mounted with fluorescent mounting medium (DakoCytomation). Histological analyses were carried out with a confocal laser microscope (LSM710, Carl Zeiss).

In murine samples, we used polyclonal Ab (pAb) against mouse CD69 (R & D) and mAb against Myl9/12 (F-6) and von Willebrand factor (Abcam). AlexaFluor 488-labeled anti-rabbit antibodies, AlexaFluor 647-labeled anti-goat antibodies, and AlexaFluor 546 anti-sheep IgG Abs from Invitrogen were used as secondary antibodies.

In human samples, we used pAb against von Willebrand factor (Abcam) and mAbs against Myl9/12 (F-6) and AlexaFluor 647-labeled human CD69 (FN50, BD). AlexaFluor 488-labeled anti-rabbit antibodies and AlexaFluor 546 anti-sheep IgG antibodies from Invitrogen were used as secondary antibodies. DAPI and CellMask Deep Red from Invitrogen were used for nucleus and cytoplasm staining. Data sets were analyzed with the ImageJ software program (National Institutes of Health, Bethesda, MD, USA). Non-inflammatory tissues from patients with colon cancer were used as a control.

To evaluate the frequency of CD69-expressing cells that are located near to either Myl9/12-positive or Myl9/12-negative vessels, we counted the number of CD69-expressing cells existed within a field (200 μm square) where Myl9/12-positive or Myl9/12-negative vessels were located in the center, and then the counted numbers were divided by the number of all DAPI-positive cells in the same field.

### The Enzyme-Linked Immunosorbent Assay (ELISA) of Human Blood Samples

The Myl9 levels in human plasma were measured by an ELISA (Human MLC2/MYL9 ELISA Kit; LifeSpan BioSciences, Inc., WA, USA) according to the manufacturer’s instructions. Human plasma samples were serially diluted and at least two points were measured.

The plasma IL-6, IL-1β, and TNFα production in mice with DSS-induced colitis was measured by ELISAs using a Mouse Il-6 ELISA kit (Abcam: ab222503), Mouse Il-1 beta ELISA kit (Abcam: ab197742), and a Mouse TNF-alpha Quantikine ELISA kit (R&D Systems: MTA00B), respectively, according to the manufacturer’s instructions.

### Statistical Analysis

All statistical analyses were performed using the GraphPad Prism software program. The data were expressed as the mean ± SEM of the corresponding numbers of samples. Statistical significance was determined using a paired or unpaired two-tailed Student’s *t*-test. A one-way analysis of variance (ANOVA) was used for multiple comparisons. Kaplan-Meier survival curves and a log-rank test were used to analyze the survival of mice treated with either anti-Myl9/12 Ab or Control Ab. Pearson’s correlation coefficient was used analyze the correlation between two parameters. *P* values of <0.05 were considered to indicate statistical significance.

## Results

### The Colonic Expression of Myl9/12 Was Increased After the Administration of DSS

**Figure 1:**
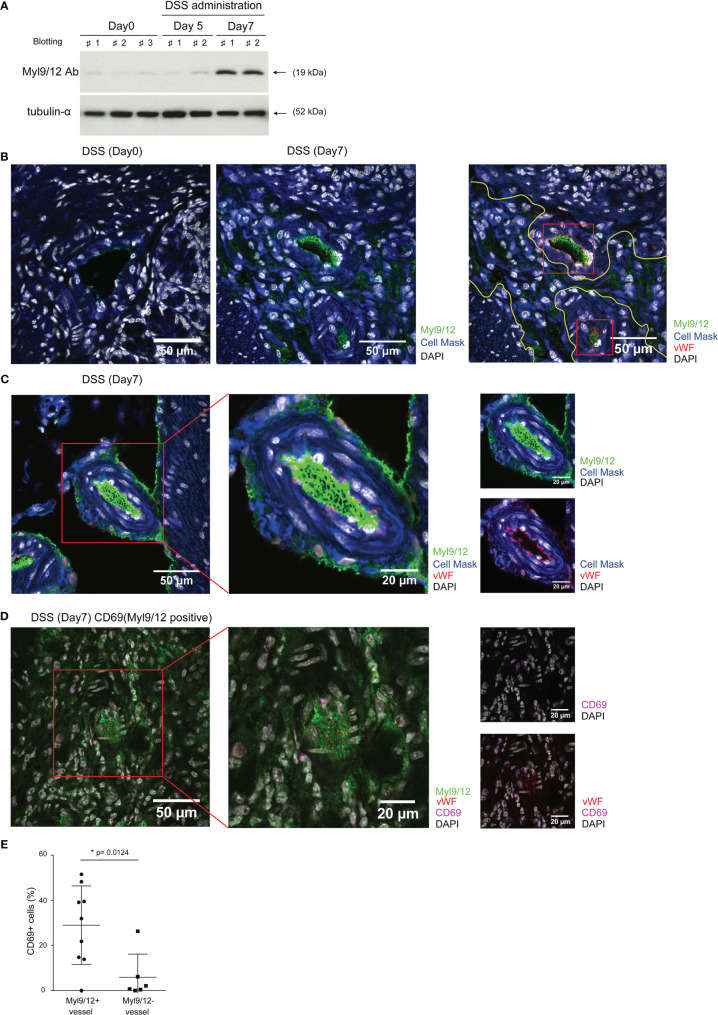
The Myl9/12 expression in the colon after the administration of DSS. **(A)** Immunoblotting of the total amount of Myl9/12 protein in the colon at 0, 5, and 7 days after the administration of DSS. Tubulin-α protein was used as a loading control. **(B–D)** The immunohistological analysis of the colon at 0 and 7 days after the administration of DSS, stained as indicated. Red square indicates a blood vessel **(C)** or CD69-expressing cells close to Myl9/12-positive vessels **(D)**. **(E)** The frequency of CD69-expressing cells located near to Myl9/12-positive vessels *versus* Myl9/12-negative vessels in inflamed colon specimens from mice with DSS-induced colitis. A total of nine fields, where Myl9/12-postive vessels were located in the center and six fields, where Myl9/12-negative vessels were located in the center were examined using three individual mice with DSS-induced colitis. *p < 0.05.

To determine the involvement of Myl9/12 in the pathogenesis of colitis, we first examined whether the Myl9/12 expression was increased under the inflammatory conditions in the colon. To this end, we used a DSS-induced colitis model (**Supplementary Figure 1A**), which was established by treating wild-type C57BL/6 mice with 2% DSS, which was administered in their drinking water for 7 days, and used an antibody that can detect Myl9, Myl12a, and Myl12b (17). Myl9/12 were hardly detected in the colon from the healthy (day 0) mice (**Figure 1A**) and the levels were still low in mice that had consumed DSS for 5 days, but were notably increased 7 days after the administration of DSS (**Figure 1A**), indicating that the Myl9/12 protein expression in the colon is increased under inflammatory conditions.

We next performed an IHC analysis using frozen tissue sections to examine where the expression of Myl9/12 was located. As expected, the Myl9/12 expression was very limited in the healthy colon (DSS, Day 0) but was strongly detected in the inflamed colon (DSS, Day 7) (**Figure 1B**). To clarify the detailed location of the Myl9/12 expression in the inflamed colon, we next visualized the blood vessels by staining with von Willebrand factor (vWF). The Myl9/12 expression was mainly detected inside blood vessels (red squares in **Figure 1B** [right] and 1C). We found that some blood vessels in the inflamed colon contained “Myl9 nets,” consisting of Myl9-formed net-like structures together with vWFs (**Figure 1C**), which was a similar structure to that previously reported in the inflamed lung (17). In addition, the Myl9/12 expression was also detected even in the parenchyma at inflammatory sites (within the yellow line in **Figure 1B** [right]).

Since Myl9/12 are functional ligands for CD69 at inflammatory sites (12, 17), we next examined whether CD69-expressing cells were located around Myl9/12-positive vessels. We calculated the percentages of CD69-expressing cells located close to either Myl9/12-positive (**Figure 1D**) or Myl9/12-negative vessels (**Supplementary Figure 1B**) and found that the percentage of CD69-expressing cells located close to Myl9/12-positive vessels was significantly higher in comparison to locations close to Myl9/12-negative vessels (**Figure 1E**). These data show that CD69-expressing cells accumulated in the inflamed colon where the expression of Myl9/12 was detected.

### The Administration of Anti-Myl9/12 Ab Ameliorated DSS-Induced Colitis

**Figure 2:**
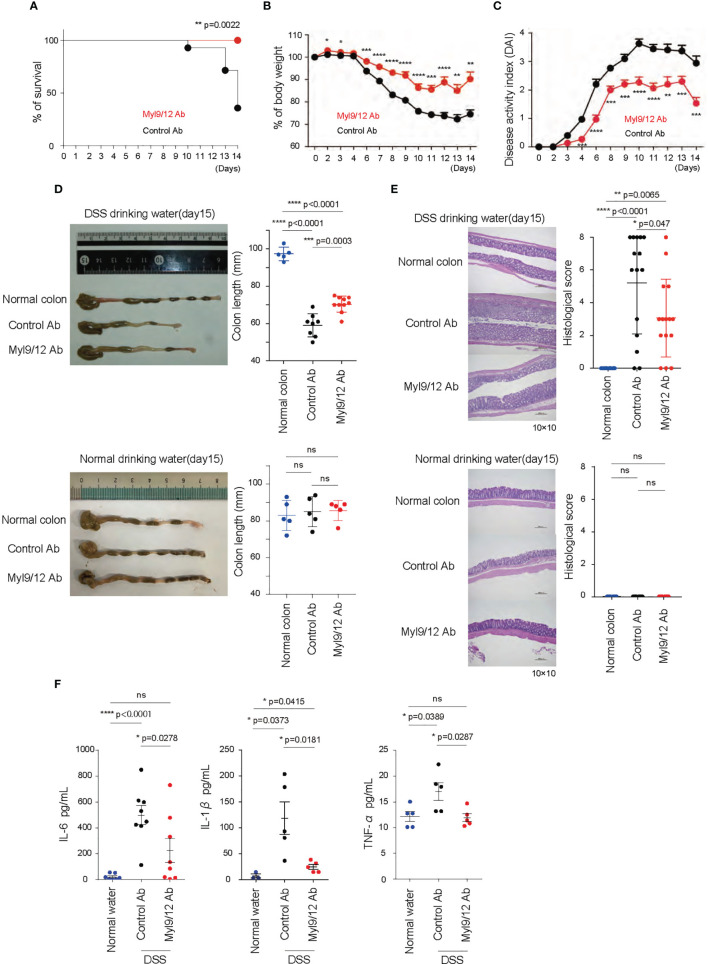
The administration of anti-Myl9/12 Ab ameliorated DSS-induced colitis. **(A–C)** The rate of survival **(A)**, percent change of body weight on each day relative to those on day 0 **(B)**, and disease activity index (DAI) **(C)** of the DSS-induced colitis model mice treated with either anti-Myl9/12 Ab or Control Ab (n = 10 per group); *p < 0.05; **p < 0.01; ***p < 0.001; ****p < 0.001. **(D)** A representative image showing the gross appearance of the colon from the DSS-induced colitis model mice (upper) and the mice drinking normal water (bottom) treated with either anti-Myl9/12 Ab or Control Ab. Each image included the image from a healthy mouse (Normal colon). The colon length from the mice either at the time of death or on day 15. ***p < 0.001; ****p < 0.001. **(E)** Hematoxylin and eosin (HE) staining of the colon from a healthy mouse or DSS-induced colitis model mice treated with either anti-Myl9/12 Ab or Control Ab (left). The histological score is determined based on the changes described in **Supplementary Table 2**. *p < 0.05; **p < 0.01; ****p < 0.001. **(F)** IL-6, IL-1β, TNFα production in the plasma from DSS-induced colitis model mice treated with either anti-Myl9/12 Ab or Control Ab. *p < 0.05; ****p < 0.0001. ns, not significant.

We next examined whether the administration of anti-Myl9/12 Ab, which is known to inhibit the interaction between Myl9/12 and CD69 (17), could ameliorate DSS-induced colitis. Either anti-Myl9/12 Ab or control Ab was injected every 2 days starting on day 0, when the mice started drinking water containing DSS (**Supplementary Figure 1C**). The mice with anti-Myl9/12 Ab treatment showed longer survival (**Figure 2A**), milder body weight loss (**Figure 2B**), and lower DAI values in comparison to those with control Ab treatment (**Figure 2C**). Furthermore, the colon length in the mice with anti-Myl9/12 Ab treatment was less reduced in comparison to that in the mice with control Ab treatment (**Figure 2D**). The colon from the mice with control Ab treatment also showed severe morphological changes, including the loss of crypts in large areas and the infiltration of inflammatory cells into the submucosa on H&E staining; in comparison, the findings in mice with anti-Myl9/12 Ab treatment were relatively mild (**Figure 2E**). In addition, treatment with control Ab or anti-Myl9/12 Ab did not show any influence in healthy mice (**Figures 2D, E**). Notably, the plasma levels of IL-6, IL-1β, and TNFα from DSS colitis mice were significantly diminished in mice that received anti-Myl9/12 Ab treatment in comparison to those that received control Ab treatment (**Figure 2F**). These data demonstrate that anti-Myl9/12 Ab treatment ameliorates DSS-induced colitis, and that anti-Myl9/12 Ab can be used as an effective treatment for colitis.

### Myl9/12 Were Highly Expressed in the Inflamed Colon in UC Patients

**Figure 3:**
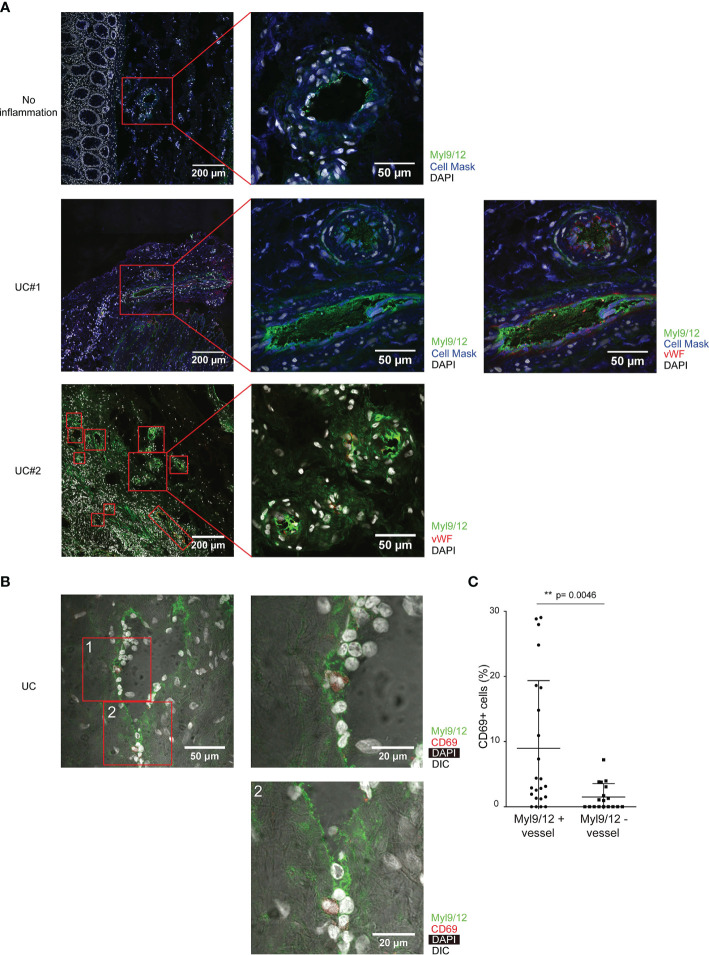
The high expression of Myl9/12 in the inflamed colon in UC patients. **(A)** Immunohistological analyses of inflamed colon specimens of UC patients and non-inflamed colon specimens of colon cancer patients, stained as indicated (UC, n = 3; No inflammation, n = 3). Red squares indicate blood vessels, which are visualized by vWF staining (×400). **(B)** Immunohistological analyses of the inflamed colon of UC patients, stained as indicated. Red squares indicate blood vessels (×400). **(C)** The frequency of CD69-expressing cells close to Myl9/12-positive vessels *versus* Myl9/12-negative vessels in the inflamed colon. A total of 23 fields where Myl9/12-postive vessels were located in the center and 18 fields where Myl9/12-negative vessels were located in the center were examined using colon samples from five patients. **p < 0.01.

We next examined the Myl9/12 expression in inflamed tissues from UC patients by IHC. The significant expression of Myl9/12 protein was detected in the colon from UC patients (**Figure 3A** middle and bottom), whereas the normal non-inflamed colonic mucosa obtained from colon cancer patients showed the limited expression of Myl9/12 protein (**Figure 3A** top). We further noted that the Myl9/12 expression was mainly localized within the blood vessels, which were visualized according to the expression of vWF (red squares in **Figure 3A** middle right and bottom). In addition, the expression of some Myl9/12 was detected in the parenchyma (the regions outside of the red square in **Figure 3A** bottom). Myl9/12 formed net-like structures, creating “Myl9 nets” within the vessels (**Figure 3A** middle and bottom).

We next examined whether CD69-expressing cells were localized close to the blood vessels expressing Myl9/12, as we did in **Figures 1D, E**. IHC showed that CD69-expressing cells were preferentially localized close to Myl9/12-positive vessels (**Figure 3B**). Furthermore, the percentage of CD69-expressing cells that were close to Myl9/12-positive vessels was significantly greater than the percentage of CD69-expressing cells that were close to Myl9/12-negative vessels (**Figure 3C**).

### The Correlation Between the Disease Severity and the Plasma Myl9 Levels of UC Patients

**Figure 4:**
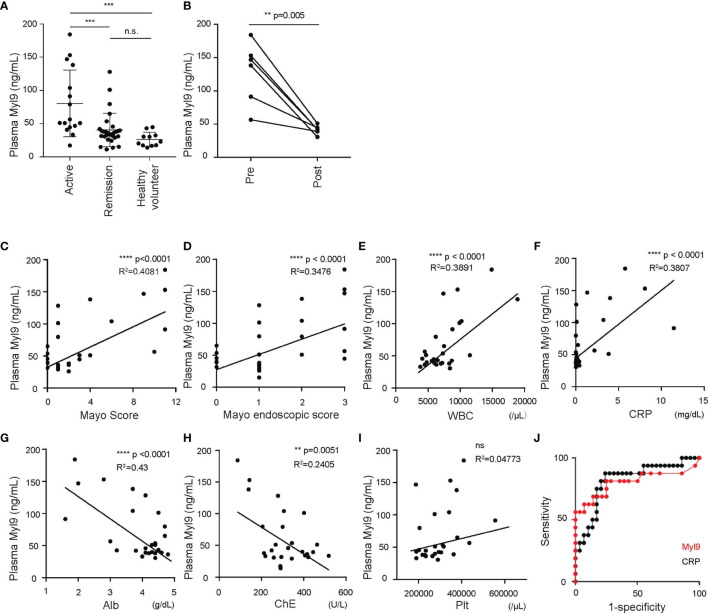
Plasma Myl9 levels depict the disease activity of UC. **(A)** Plasma Myl9 levels from UC patients with active UC (Mayo ≥3) or UC patients who were in remission (Mayo <2) and healthy volunteers (active UC, n = 16, remission, n = 29, healthy volunteers, n = 11). **p < 0.01; ***p < 0.001; ****p < 0.001. **(B)** The plasma Myl9 levels in the patients before (pre) and after (post) treatment. The Myl9 levels from the same patients are connected. **(C–J)** The correlations of plasma Myl9 with the Mayo Score **(C)**, Mayo endoscopic score **(D)**, WBC count **(E)**, CRP **(F)**, serum Alb **(G)**, serum cholinesterase **(H)**, and platelet count **(I)**. **(J)** ROC curves for Myl9 and CRP in UC patients in remission and those with active UC. ns, not significant.

We next examined the utility of the Myl9 levels in the plasma as a biomarker for UC. We used an ELISA system that can detect Myl9 in plasma. We found that the Myl9 levels in the plasma from patients with active UC (Mayo score ≥3) were significantly higher than those in UC patients who were in remission (Mayo score <2) and in healthy volunteers (**Figure 4A**). Furthermore, the plasma Myl9 levels were significantly decreased upon treatment in the same patients (**Figure 4B**), suggesting that plasma Myl9 levels may depict disease severity.

Accordingly, we next examined whether the plasma Myl9 level showed any correlation with other clinical data. First, the plasma Myl9 levels were well correlated with both the Mayo score, commonly used to describe the general status of UC patients, and the Mayo endoscopic score, commonly used to describe the endoscopic status (24) (**Figures 4C, D**), showing that the plasma Myl9 level reflects the disease activity. Furthermore, we found that the C-reactive protein (CRP) level and white blood cell (WBC) count, both clinical markers indicating levels of inflammation that are commonly used in practice, were also positively correlated with the plasma Myl9 level (**Figures 4E, F**). In contrast, the levels of albumin (Alb) and cholinesterase (ChE) were negatively correlated with the plasma Myl9 levels (**Figures 4G, H**). Given that UC patients suffer from a deteriorated nutritional status according to the severity of bowel disease, these negative correlations also indicate the disease severity. Interestingly, the platelet level in the blood was not correlated with the plasma Myl9 levels (**Figure 4I**), although plasma Myl9 seems to be derived from platelet activation (12). We think that the local activation of platelets in the inflamed colon may release Myl9, resulting in high concentrations of systemic Myl9 in the plasma of patients with active UC.

Plasma CRP levels are commonly used to evaluate inflammation in the body, and our data showed that the plasma CRP levels were well-correlated with the disease severity of UC (**Supplementary Figures 2A–H**). To determine the sensitivity and specificity of plasma Myl9 in predicting the disease status of UC patients, we generated receiver operating characteristics (ROC) curves and compared them to the curves for the plasma CRP levels (**Figure 4J**). The area under the curve (AUC) for plasma Myl9 was 0.819, whereas that for plasma CRP was 0.8047 (**Figure 4J**). The cut-off value of plasma Myl9 was 40.89 ng/ml (sensitivity = 87.5%, specificity = 75.86%). These data suggest that the plasma Myl9 level may predict active UC more accurately (or at least similarly) than the plasma CRP level.

### The Myl9/12 Expression in CD Patients

**Figure 5:**
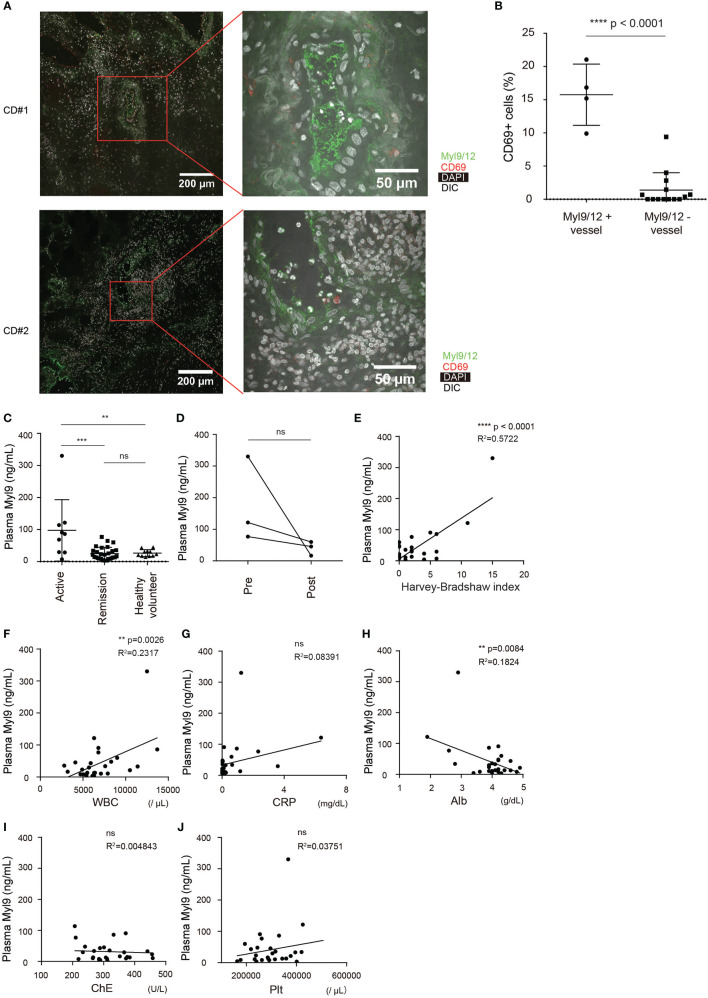
The Myl9/12 expression in CD patients. **(A)** Immunohistological analyses of inflamed gut specimens from CD patients, stained as indicated (n = 3). Red squares indicate blood vessels, which are identified based on the DIC image (×400). **(B)** The frequency of CD69-expressing cells close to Myl9/12-positive vessels *versus* Myl9/12-negative vessels in the inflamed gut. A total of 4 fields where Myl9/12-postive vessels were located in the center and 14 fields where Myl9/12-negative vessels were located in the center were examined in gut samples from three patients. **(C)** Plasma Myl9 levels from CD patients with active CD (HBI ≥5) or CD patients who were in remission (HBI <5) and healthy volunteers (active CD, n = 9, remission CD, n = 28, healthy volunteers, n = 11). **p < 0.01; ***p < 0.001; ****p < 0.001. **(D)** The plasma Myl9 levels in the patients before (pre) and after (post) treatment. The Myl9 levels from the same patients are connected. **(E–J)** Correlations of the plasma Myl9 level with the HBI **(E)**, WBC count **(F)**, CRP **(G)**, serum Alb **(H)**, serum cholinesterase **(I)**, and platelet count **(J)**. ns, not significant.

We next examined whether Myl9/12 are also involved in the pathogenesis of CD. Inflamed gut specimens were surgically obtained from CD patients, and the Myl9/12 expression was examined by IHC analysis. We found that the inflamed gut from CD patients strongly expressed Myl9/12, mainly within the blood vessels (red squares in **Figure 5A**), and some formed “Myl9 nets” within the vessels (**Figure 5A**, right). Furthermore, the percentage of CD69-expressing cells located close to Myl9/12-positive vessels was significantly greater than that in cells located close to Myl9/12-negative vessels, indicating that CD69-expressing cells were preferentially located close to Myl9/12-positive vessels (**Figure 5B**).

We also examined whether the plasma Myl9 level was correlated with the disease activity of CD. We found that the plasma Myl9 levels in patients with active CD were significantly higher than those in patients in remission and healthy volunteers (**Figure 5C**). The plasma Myl9 levels decreased upon treatment in the same patients (**Figure 5D**); however, this result did not reach statistical significance due to the limited availability of samples from CD patients.

Importantly, the plasma Myl9 level was positively correlated with the Harvey-Bradshaw Index (HBI), which determines the current severity of CD; patients with a high HBI value of ≥5 are considered to have active CD (**Figure 5E**). In addition, plasma Myl9 level was positively and negatively correlated with the WBC count and the Alb level, respectively, although the correlation coefficients were small (**Figures 5F, H**). In contrast, there was no obvious correlation between plasma Myl9 and CRP, ChE, or Plt (**Figures 5G, I, J**). These data show that there is only a mild correlation between plasma Myl9 and the disease severity of CD, and that the correlations in CD are much weaker than those in UC.

## Discussion

In the present study, we detected Myl9/12 protein very easily in the inflamed gut, not only in mice with colitis induced by the administration of DSS, but also in UC and CD patients. Anti-Myl9/12 Ab treatment of mice with DSS-induced colitis prolonged their survival, and reduced the loss of body weight and DAI in comparison to mice with control Ab treatment. These findings suggest that anti-Myl9/12 Ab is a new therapeutic target for IBD. Importantly, the plasma Myl9 levels in IBD patients, especially UC, were positively correlated with their disease severity, suggesting that plasma Myl9 is a viable new biomarker indicating the severity of disease activity of IBD, especially in patients with UC.

In 2016, we first reported that Myl9, Myl12a, and Myl12b are functional ligands for CD69 (17). Myl9 is produced by activated platelets and often creates “Myl9 nets” at sites of inflammation. CD69-expressing inflammatory leukocytes are recruited into or maintained in the inflamed tissues through interaction with the Myl9 nets, resulting in the exacerbation of inflammatory diseases (12, 18). IBD is a well-recognized inflammatory disorder, the pathogenesis of which requires the recruitment of inflammatory leukocytes, especially T cells, to the gastrointestinal tract (8). In the present study, we detected the high expression of Myl9/12 in inflamed tissues from both UC and CD patients, as well as in mice with DSS-induced colitis, and CD69-expressing cells were preferentially located close to Myl9/12-positive vessels at the inflammatory sites. Furthermore, anti-Myl9/12 Ab treatment of mice with DSS-induced colitis ameliorated the inflammation, indicating that Myl9/12 regulates the pathogenesis of IBD. These data were consistent with our previous findings in which CD69 KO mice were strongly protected against DSS-induced colitis (14). It is also possible that CD69-independent mechanisms contribute to the inhibitory effect of anti-Myl9/12 Ab treatment in DSS-induced colitis. Further careful experiments are required to elucidate the requirement of CD69 in the pathogenesis of IBD.

Since Myl9 nets created at the inflamed site are most likely derived from activated platelets, we think that whatever the reason (environmental factors or a genetic predisposition), epithelial barrier defects or dysregulated immune responses induce the activation of the coagulation system and thereby activate platelets at the local site. The activated platelets then produce Myl9 and create Myl9 nets, which are used to recruit CD69-expressing inflammatory cells to inflamed tissues and induce their accumulation, leading to the exacerbation of inflammation.

Notably, we found that the plasma Myl9 level had a strong positive correlation with the disease severity in IBD patients, especially in UC patients. The plasma Myl9 levels were high in patients with active UC but low in those in remission and healthy volunteers, demonstrating that plasma Myl9 accurately depicts the disease severity in UC patients. Furthermore, the plasma Myl9 levels were significantly reduced following various treatments (e.g., surgical operations, cytoapheresis, and the administration of prednisolone, anti-TNF Ab and calcineurin inhibitors or other biologics and small molecules) within the same patients. A decrease in plasma Myl9 was associated with remission but not with the administration of treatment, demonstrating that plasma Myl9 levels are a useful biomarker for evaluating the disease activity in UC patients. While CRP is the plasma marker most commonly used to evaluate the disease activity in patients with inflammatory disease (25), our analysis using an ROC curve and the AUC indicated that the plasma Myl9 level depicted the disease activity in UC patients with superior sensitivity and specificity to plasma CRP. In contrast, the correlation between plasma Myl9 and the disease activity in CD patients was much milder than that in UC patients. Because UC patients in general have more severe inflammation over a wider area of colon than CD patients, we think that plasma Myl9 reflects the degree of inflammation.

Current treatments, including 5-aminosalicylic acid (5-ASA), corticosteroids, azathioprine, and biologics such as infliximab and adalimumab (anti-TNF Ab), vedolizumab (anti-Integrinα4β7 Ab), and ustekinumab (anti-IL-12/23p40 Ab), have been used for both UC and CD patients. Calcineurin-inhibitor and tofacitinib (JAK inhibitor) have been used for UC patients. While the increase in the use of these drugs has resulted in a reduction of the rate of surgery, some patients suffering from intractable inflammation must still undergo surgery (26, 27), which reduces the quality of life (QOL). Furthermore, as immunosuppressive agents, biologics are associated with a high risk of infectious complications and malignancy, which may increase the risk of mortality (28–30). Accordingly, new treatments for IBD are always desired. Notably, our data showed that anti-Myl9/12 Ab treatment ameliorated DSS-induced colitis, and our previous study showed that anti-CD69 Ab treatment ameliorated DSS-induced colitis (14). These data suggest that both ant-Myl9/12 and anti-CD69 Abs may have therapeutic potential for IBD. Further studies will be required to determine whether combination therapy with anti-Myl9/12 or anti-CD69 Abs and current treatments will improve the outcomes of IBD patients.

In the present study, we showed that Myl9/12 molecules are involved in the pathogenesis of IBDs, such as UC and CD, and that the plasma Myl9/12 level depicts the disease severity of IBD, especially in patients with UC, suggesting that the plasma Myl9/12 level may be a useful biomarker for IBD. Furthermore, the blockade of the interaction between CD69 and Myl9/12 using specific Abs against Myl9/12 and CD69 ameliorated the inflammation. We conclude that anti-Myl9/12 and anti-CD69 Abs may have therapeutic applications in the management of patients with intractable IBD.

## Data Availability Statement

The original contributions presented in the study are included in the article/**Supplementary Material**. Further inquiries can be directed to the corresponding authors.

## Ethics Statement

The studies involving human participants were reviewed and approved by the Ethics Committee of the Chiba University Graduate School of Medicine (No. 898). The patients/participants provided their written informed consent to participate in this study. The animal study was reviewed and approved by Chiba University.

## Author Contributions

MY and MYK designed the study, performed experiments, and analyzed data. MY, MYK, and TosN wrote the manuscript. TI, KH, YE, YSW, and RY performed experiments and analyzed the data. TomN provided clinical samples. NK, HM and TosN provided helpful advice. All authors contributed to the article and approved the submitted version.

## Funding

This work was supported by the following grants: Ministry of Education, Culture, Sports, Science and Technology (MEXT Japan) Grants-in-Aid for Scientific Research (S) 26221305, JP19H05650, Young Scientists (B) 17K15715, Challenging Research (Exploratory) 18K19466, Practical Research Project for Allergic Diseases and Immunology (Research on Allergic Diseases and Immunology) from Japan Agency for Medical Research and Development, AMED (Nos. JP19ek0410060, JP20ek0410060); AMED-CREST, AMED (Nos. JP18gm1210003, JP19gm1210003, JP20gm1210003), The Uehara Memorial Foundation, Astellas Foundation for Research on Metabolic Disorders, and Takeda Science Foundation.

## Conflict of Interest

The authors declare that the research was conducted in the absence of any commercial or financial relationships that could be construed as a potential conflict of interest.

## Supplementary Material

Supplementary Figure 1 A schematic illustration of the DSS-induced colitis model. **(A)** Colitis was induced in female mice (age, 7 to 8 weeks) by the addition of 2% DSS to drinking water that had been purified by filtration. On days 5 and 7, mice were sacrificed for the indicated analyses. **(B)** The immunohistological analysis of the colon at 7 days after the administration of DSS, stained as indicated. CD69-expressing cells and Myl9/12-negative vessel. **(C)** Colitis was induced in female mice (age, 7 to 8 weeks) by adding 2% DSS to drinking water that had been purified by filtration. Anti-Myl9/12 mAb (114-2G9) or isotype Ab (104-6G4) was intraperitonially injected into the mice every 2 days from day 0 to day 12 (day 0: 200 µg, day 2–12: 100 µg, respectively). On day 15, the mice were sacrificed for the analysis.

Supplementary Figure 2 The correlation between plasma CRP levels and disease activity of UC. **(A)** Plasma CRP levels from UC patients with active UC (Mayo ≥3) or patients with UC in remission (Mayo <2) and healthy volunteers (active UC, n = 13, remission UC, n = 29, healthy volunteer, n = 11). *p < 0.05; **p < 0.01; ***p < 0.001. **(B)** The plasma CRP levels in the patients before (pre) and after (post) treatment. The CRP levels from the same patients were connected. **(C–H)** The correlations of the plasma CRP levels with the Mayo score **(C)**, Mayo endoscopic score **(D)**, WBC count **(E)**, serum Alb **(F)**, serum cholinesterase **(G)**, and platelet count **(H)**.

Supplementary Table 1 Scoring of the disease activity index in a DSS-induced colitis mouse model. Normal stool, well-formed pellets; loose stool, pasty and semi-formed stool that does not stick to the anus; diarrhea, liquid stool that sticks to the anus.

Supplementary Table 2 The histological scores in the DSS-induced colitis mouse model.
