# Supplementary figures and images for: Myosin Light Chain 9/12 Regulates the Pathogenesis of Inflammatory Bowel Disease
Source: Front Immunol. 2021 Jan 29;11:594297. doi: 10.3389/fimmu.2020.594297 (PMC7878395; doi:10.3389/fimmu.2020.594297)

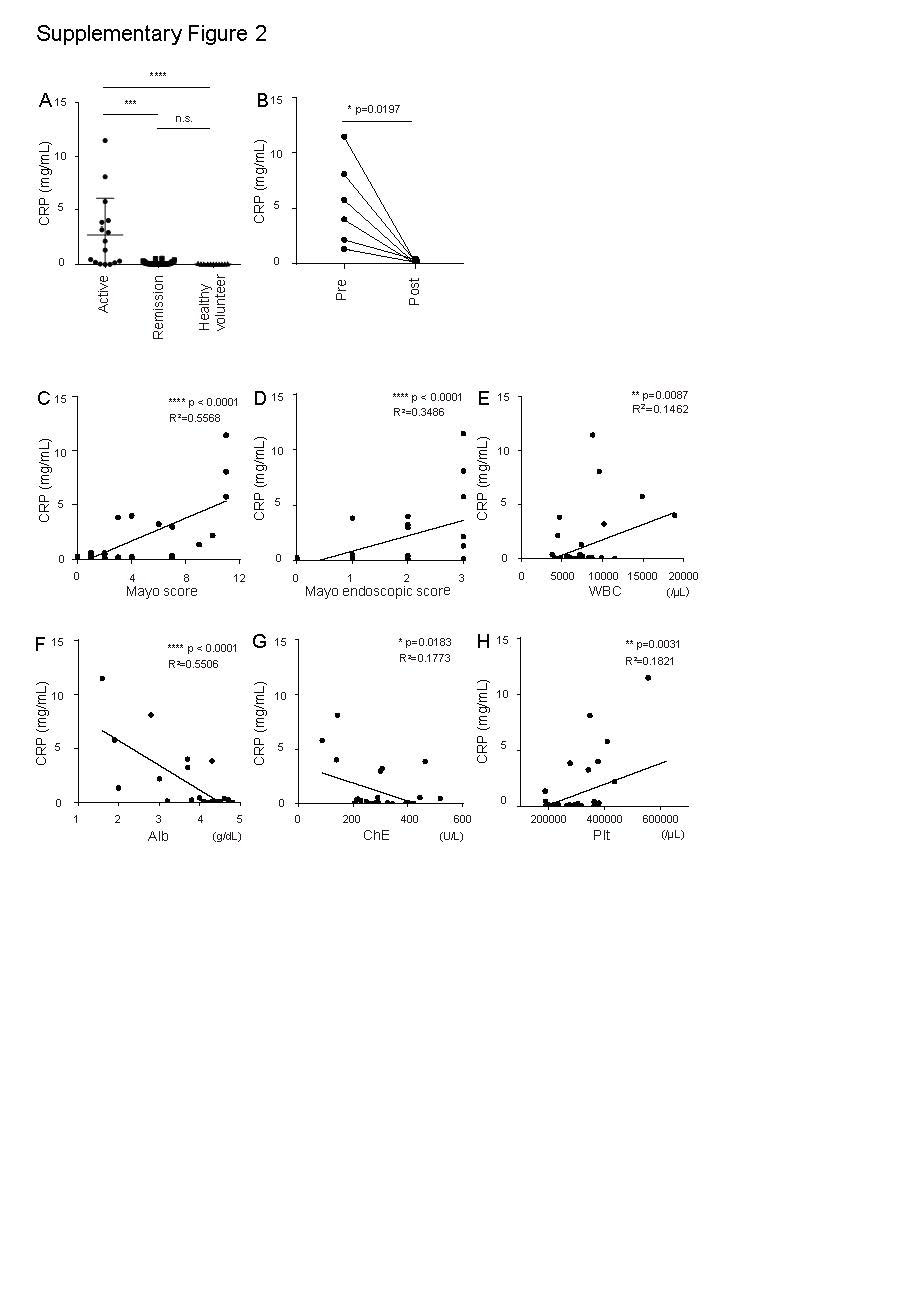

Supplement: Supplementary Figure 2 — The correlation between plasma CRP levels and disease activity of UC. (A) Plasma CRP levels from UC patients with active UC (Mayo ≥3) or patients with UC in remission (Mayo <2) and healthy volunteers (active UC, n = 13, remission UC, n = 29, healthy volunteer, n = 11). *p < 0.05; **p < 0.01; ***p < 0.001. (B) The plasma CRP levels in the patients before (pre) and after (post) treatment. The CRP levels from the same patients were connected. (C–H) The correlations of the plasma CRP levels with the Mayo score (C), Mayo endoscopic score (D), WBC count (E), serum Alb (F), serum cholinesterase (G), and platelet count (H). [file Image_2.jpeg]
